# Relationship between caffeine intake and autosomal dominant polycystic kidney disease progression: a retrospective analysis using the CRISP cohort

**DOI:** 10.1186/s12882-018-1182-0

**Published:** 2018-12-27

**Authors:** Katelyn A. McKenzie, Mirelle El Ters, Vicente E. Torres, Peter C. Harris, Arlene B. Chapman, Michal Mrug, Frederic F. Rahbari-Oskoui, Kyongtae Ty Bae, Douglas P. Landsittel, William M. Bennett, Alan S. L. Yu, Jonathan D. Mahnken

**Affiliations:** 1 Department of Biostatistics, University of Kansas Medical Center, Mail Stop 1026, 3901 Rainbow Blvd., Kansas City, KS 66160 USA; 2 Division of Nephrology and Hypertension, Mayo Clinic, Rochester, MN USA; 3 Section of Nephrology, University of Chicago School of Medicine, Chicago, IL USA; 4 Division of Nephrology, University of Alabama and the Department of Veterans Affairs Medical Center, Birmingham, AL USA; 5 Department of Internal Medicine, Emory University School of Medicine, Atlanta, GA USA; 6 Department of Radiology, University of Pittsburgh School of Medicine, Pittsburgh, PA USA; 7 Department of Biomedical Informatics, University of Pittsburgh School of Medicine, Pittsburgh, PA USA; 8 Legacy Good Samaritan Hospital, Portland, OR USA; 9 Division of Nephrology and Hypertension and the Jared Grantham Kidney Institute, University of Kansas Medical Center, Kansas City, KS USA

**Keywords:** Caffeine, CRISP, ESRD, Linear mixed models, Polycystic kidney disease

## Abstract

**Background:**

Caffeine has been proposed, based on in vitro cultured cell studies, to accelerate progression of autosomal dominant polycystic kidney disease (ADPKD) by increasing kidney size. Since ADPKD patients are advised to minimize caffeine intake, we investigated the effect of caffeine on disease progression in the Consortium for Radiologic Imaging Studies of Polycystic Kidney Disease (CRISP), a prospective, observational cohort study.

**Methods:**

Our study included 239 patients (mean age = 32.3 ± 8.9 ys; 188 caffeine consumers) with a median follow-up time of 12.5 years. Caffeine intake reported at baseline was dichotomized (any vs. none). Linear mixed models, unadjusted and adjusted for age, race, sex, BMI, smoking, hypertension, genetics and time, were used to model height-adjusted total kidney volume (htTKV) and iothalamate clearance (mGFR). Cox proportional hazards models and Kaplan-Meier plots examined the effect of caffeine on time to ESRD or death.

**Results:**

Caffeine-by-time was statistically significant when modeling ln(htTKV) in unadjusted and adjusted models (*p* <  0.01) indicating that caffeine consumers had slightly faster kidney growth (by 0.6% per year), but htTKV remained smaller from baseline throughout the study. Caffeine consumption was not associated with a difference in mGFR, or in the time to ESRD or death (*p* > 0.05). Moreover the results were similar when outcomes were modeled as a function of caffeine dose.

**Conclusion:**

We conclude that caffeine does not have a significant detrimental effect on disease progression in ADPKD.

**Electronic supplementary material:**

The online version of this article (10.1186/s12882-018-1182-0) contains supplementary material, which is available to authorized users.

## Background

Autosomal dominant polycystic kidney disease (ADPKD) is a systemic disease that primarily affects the kidneys. ADPKD occurs in both sexes, all races and the majority of cases are caused by a genetic mutation in one of two genes, *PKD1* and *PKD2* [1]. ADPKD is the most common inherited kidney disease. This disease causes irreversible kidney damage that begins in utero and eventually leads to end stage renal disease (ESRD), of which it is a major contributor [2]. While the incidence of ADPKD is estimated to be between 1:400–1:1000, there are no curative treatments [3] and one pharmacologic therapy, tolvaptan, that was recently approved in the United States [4].

Mechanistically, these genetic mutations lead to an abnormal response to high levels of 3′:5′-cyclic adenosine monophosphate (cAMP) when intracellular levels of calcium are low. This has two effects. First, an increase in cAMP will lead to activation of the ERK signaling pathway which ultimately causes an increase in cellular proliferation. Second, an increase in cAMP will lead to activation of the cystic fibrosis transmembrane conductance regulator (CFTR), which causes an increase in chloride secretion and thus water into the cysts [5]. Additionally, while the exact role of vasopressin in ADPKD progression is not clear, it is known that vasopressin V2 receptor antagonists reduces the rate of kidney growth in ADPKD [6].

Due to the high proportion of patients who reach ESRD, it is important to understand the role of environmental factors, such as diet and other lifestyle exposures, in disease progression. Of interest is the role of caffeine. It is well known that caffeine increases cAMP levels by inhibiting phosphodiesterase, an enzyme that hydrolyzes cAMP. In 2002, Belibi et al. examined the effects of caffeine on ADPKD cyst epithelial cells in vitro [7]. They found that caffeine increased intracellular cAMP levels and potentiated the effect of desmopressin, a vasopressin analog, on chloride secretion and ERK activation. On the basis of this single, in vitro study, and on the predicted effects of increased cAMP to accelerate cell proliferation, fluid secretion, and hence kidney cyst growth, most physicians advise their patients who have ADPKD to limit caffeine intake [8].

This recommendation is open to question. In the Han:SPRD rat model of ADPKD, chronic caffeine intake to the age of 6 months did not accelerate kidney or cyst growth or the decline in GFR, although it did exacerbate hypertension [9]. There are limited studies on the effects of caffeine intake in patients with ADPKD. In 2012, Vendramini et al. studied the effects of caffeine on patients with ADPKD in a small cross-sectional study and found that renal volume, as measured by ultrasound, was not associated with caffeine intake [10]. In 2017, Girardat-Rotar et al. examined the association of coffee intake with ADPKD progression, as measured by height adjusted total kidney volume (htTKV) and glomerular filtration rate (GFR) in a prospective longitudinal study of 151 patients followed for a median of 4 years, and concluded that coffee consumption was not a significant risk factor [11].

Although suggestive evidence is beginning to surface that caffeine may not contribute to disease progression, the goal of this analysis was to examine the effects of caffeine on ADPKD progression over a longer time period in a well-studied cohort of patients with ADPKD.

## Methods

### CRISP study design and participants

Data were taken from the Consortium for Radiologic Imaging Studies of Polycystic Kidney Disease (CRISP) study, which comprised of four clinical centers, University of Alabama, Emory University, University of Kansas and the Mayo Clinic. Patients were eligible to be enrolled in the CRISP study if they were diagnosed with ADPKD, had a creatinine clearance of at least 70 mL/min, serum creatinine level of either ≤1.6 mg/deciliter for men or ≤ 1.4 mg/deciliter for women, and were between 15 and 46 years of age at baseline. Patients were excluded from the study if they had any comorbidities that would affect kidney function besides hypertension. At each visit, TKV was determined from coronal T1- and T2-weighted MRI using a stereologic method, [12–14] and corrected for height (htTKV, ml/m). GFR was measured by iothalamate clearance and indexed to body surface area (ml/min/1.73 m^2^). Lifestyle exposures, including caffeine consumption and smoking status, were collected via written surveys in the form of closed questions (Additional file 1). Patients were screened for mutations in the *PKD1* and *PKD2* genes. Further details about the CRISP study have been previously published [13, 15].

### Outcome measures

Two outcomes were used to assess disease progression. The primary outcome was height-adjusted total kidney volume (htTKV) [16]. Because kidney volume increases exponentially over time, htTKV was natural log transformed. The second outcome was measured glomerular filtration rate (mGFR) calculated by iothalamate clearance [17]. We chose to have the primary outcome be htTKV because htTKV has been shown to be a good proxy for disease state [15] and the secondary outcome to be mGFR because mGFR is used to estimate kidney filtering capabilities and thus kidney function.

### Explanatory measure of interest

Caffeine consumption at baseline was assessed as the number of cups of coffee/tea, and the number of glasses of other caffeinated beverages consumed per day, averaged over the prior month. For the primary analysis, the exposure variable of caffeine consumption was dichotomized (any vs. none). For further investigation of this relationship, we treated caffeine dose both as a continuous variable, and binned into daily quartiles of caffeine intake of 0 mg (*n* = 51), > 0-86 mg (*n* = 50), > 86-181 mg (*n* = 47), > 181-301 mg (*n* = 45) and > 301 mg (*n* = 46). To obtain a quantitative estimate of caffeine dose, each source was converted into milligrams via the following conversions: 95 mg of caffeine = 1 cup of coffee/tea (8 oz.) and 43 mg of caffeine = 1 glass of other caffeinated beverage (12 oz.) [18, 19]. When converting caffeine consumption into milligrams, if the patient was missing either (but not both) number of cups of coffee or number of other caffeinated beverages per day, it was assumed they drank none (*n* = 9). Patients missing both values at baseline (*n* = 16) were imputed to have no caffeine intake.

### Additional covariates

Additional baseline variables that were included as covariates in the multivariable adjusted models were age, race, sex, BMI, smoking, hypertension, and genetic profile. The patient’s race was categorized as either “White” or “Other” due to the small sample sizes of subsets of the other races. Based on the current understanding of the prognostic significance of ADPKD mutations, we classified the mutation data into three subgroups of gene types: truncating *PKD1*, non-truncating *PKD1*, and *PKD2* + no mutation detected (NMD) [20, 21].

### Statistical analysis

Descriptive analyses for continuous variables were expressed as means (± standard deviations) and for categorical variables were expressed as frequencies and relative frequencies. If a continuous variable did not appear to be normally distributed by its quantile-quantile (QQ) plot and histogram, the median and inter-quartile range (IQR) was reported as well. Two-sampled t-tests and Pearson’s chi square test for continuous and categorical variables, respectively, were used to assess differences in measures between caffeine and non-caffeine consumers among our sample. QQ plots and histogram residuals were used to check for assumption of normality of the two-sampled t-tests. If the assumptions for the t-tests were violated, the Wilcoxon rank sum test was used. Expected cell counts were used to assess assumptions for Pearson’s chi square test. When computing the median follow-up time, the last available time was used. Four patients had 4 follow-up visits during CRISP I without times or dates, so we imputed their follow-up time to be 3 years as these all corresponded to the year three visit.

The main statistical method utilized in this analysis was a linear mixed effect model (LME) with random intercepts. LMEs were utilized because the CRISP study has more than one measurement of all outcome measures for each patient collected over 14 years. Thus we used random effects that allowed each patient to have their own intercept parameter (i.e., the random effect). All other covariates were fixed effects. Single factor association models for each variable were first computed with adjustment only for time. Then, multivariable models, adjusting for age, sex, race, BMI, smoking, hypertension, gene type and time, were computed for each outcome variable (referred to as Model 1 for ln(htTKV) and Model 1 for mGFR). Lastly, models were adjusted for caffeine, time and their interaction for each outcome variable (referred to as Model 2 for ln(htTKV) and Model 2 for mGFR). The coefficient (β) for the effect of the interaction between caffeine and time on the outcome of ln(htTKV) represents the effect of caffeine consumption on the slope of ln(htTKV) over time. The effect size, as measured by the constant, annual difference in percentage growth of htTKV (without log-transformation) in caffeine-consumers compared to non-caffeine consumers, was calculated by taking (*e*^β^)*100% [22]. Additional file 2: Table S1 summarizes the models used in this study. Model assumptions were checked using QQ plots and plots of residuals, with appropriate adjustments for model violations such as log transformations using the natural log function. Multivariable models using daily caffeine dose as continuous and multicategory variables were centered and used in sensitivity analyses, as were models which excluded subjects missing both caffeine exposure variables (cups of coffee/tea and glasses of other caffeinated beverages per day). In some cases identified as missing visits, subjects contributed some of their study measures, just not all. Linear contrasts of Model 2 for ln(htTKV) were performed to quantify the effect of caffeine over time. Kaplan Meier plots with right-censored data were used to examine the effect of caffeine on time until ESRD or death, along with corresponding log-rank tests to compare survival between caffeine groups (any vs. none). Cox proportional hazard regression analysis was performed to examine the effects of age, sex, race, BMI, smoking, hypertension, gene type and caffeine. Statistical significance was accepted if *p* <  0.05. R (Vienna, Austria) was used for data processing and analysis.

## Results

### Patient characteristics

In 2001, 241 patients were enrolled in the CRISP study and were evaluated until 2015. Of these 241 patients, we excluded 2 because of missing genetic information, thus giving us a sample size of 239 patients. Additionally, 16 patients were missing data for both caffeine sources (coffee/tea and other caffeinated beverages) at baseline. We computed models based on the inclusion of these patients, with their caffeine intake assumed to be 0 mg, then performed additional sensitivity analyses in which these patients were excluded. The median follow-up time was 12.5 years (IQR: 8.7,13.0). The minimum number of patient visits was 4 and the maximum number of visits was 8 (25th, 50th and 75th percentiles were 6, 8 and 8, respectively).

At baseline, the average (± standard deviation [sd]) and median (IQR) age of our sample was 32.3 (±8.7) and 33.8 (25.1, 39.7) years, respectfully. Sixty percent of our patients were female and 87% were white. 61% of our patients had hypertension at baseline and 17% of our patients reported smoking. The majority of our patients had a truncating mutation in the *PKD1* gene (53%) while 25% of the patients had a non-truncating mutation in *PKD1* and 22% had either a mutation in *PKD2* or no mutations detected (NMD) in either *PKD1* or *PKD2*. The mGFR was relatively preserved, as the average (± sd) and median (IQR) values were 97.7 (±24.8) and 94.7 (78.8, 114.8) mL/min/1.73m^2^, respectively. At baseline, the average (± sd) and median (IQR) of httkv in our sample was 621.6 (±373.9) and 504.4 (350.2, 773.9) ml/m, respectfully.

At baseline, 79% of the patients reported consuming caffeine. The minimum amount of caffeine consumed was 0 mg/day and the maximum was 1425 mg/day (25th, 50th and 75th percentiles = 29.3, 129, and 233 mg/day, which would be equivalent to 0.31, 1.36 and 2.45 eight-ounce cups of coffee per day, respectively). Table 1 shows the baseline characteristics of caffeine consumers compared to non-caffeine consumers. While there were no statistically significant differences between baseline characteristics, 19% of caffeine consumers reported smoking at baseline compared to 8% of non-caffeine consumers (*p*-value = 0.088). Caffeine consumers had a slightly lower median (IQR) baseline htTKV compared to non-caffeine consumers [479.9 (342.4, 726.9) mL/m compared to 567.5 (382.6, 872.7) mL/m; p-value = 0.22] and slightly higher mGFR [96.4 (78.7, 114.7) mL/min/1.73m^2^ compared to 88.7 (79.1, 114.8) mL/min/1.73m^2^; *p*-value = 0.36) but neither of these were statistically significant. At the time of this analysis, 42 patients had reached end-stage renal disease (ESRD) and 3 patients died, while 194 had not reached ESRD or were censored.

**Table 1:** Demographics and estimates of kidney function in the CRISP population at baseline

Characteristic		Total *N* = 239	Caffeine Consumer *N* = 188 (79)	Non-Caffeine Consumer *N* = 51 (21)	*P*-value for difference between caffeine and non-caffeine consumers
Age*, years	Mean (± sd)	32.3 ± 8.7	32.3 ± 8.9	32.4 ± 9.0	0.935
	Median (IQR)	33.8 (25.1, 39.7)	33.6 (25.0, 39.7)	34.2 (16.0, 39.4)	
Race (White), n (%)		207 (87)	167 (89)	40 (78)	0.089
Sex (Male), n (%)		96 (40)	78 (41)	18 (35)	0.523
BMI*, kg/m^2^	Mean (± sd)	25.91 ± 5.3	26.1 ± 5.5	25.3 ± 4.3	0.564
	Median (IQR)	25.17 (21.98, 28.64)	25.24 (22.04, 29.03)	25.07 (22.06, 27.64)	
Smoking, n (%)		40 (17)	36 (19)	4 (8)	0.088
Hypertension, n (%)		146 (61)	113 (60)	33 (65)	0.663
Genotype, n (%)					
PKD1 + truncation		127 (53)	100 (53)	27 (53)	
PKD1 + no truncation		60 (25)	49 (26)	11 (22)	0.691
PKD2 + NMD		52 (22)	39 (21)	13 (25)	
htTKV*, ml/m	Mean (± sd)	621.6 ± 373.9	606.6 ± 367.7	683.7 ± 403.5	0.222
	Median (IQR)	504.4 (350.2, 773.9)	479.9 (342.4, 726.9)	567.5 (382.6, 872.7)	
Iothalamate Clearance*^ϕ^, ml/min/1.73m^2^	Mean (± sd)	97.7 ± 24.8	98.2 ± 24.6	96.9 ± 25.2	0.358
	Median (IQR)	94.70 (78.78, 114.75)	96.36 (78.74, 114.72)	88.71 (79.05, 114.83)	

Values are given for the total population and based on caffeine intake. *P-value based on Wilcoxon rank sum test due to violation of t-test assumptions. ^ϕ^*N* = 234 (5 values missing at baseline but contributed subsequent measures for mixed model results). NMD refers to no mutation detected in the genetic analysis

### Association of caffeine intake with kidney volume and GFR over time

Linear mixed models were first fit for each covariate separately to determine each single factor association over time (eg. baseline age and time as fixed effects in the first model, baseline hypertension and time as fixed effects in the second model, etc., see Additional file 2: Table S2). When using ln(htTKV) as the outcome variable, age, BMI, hypertension, race and gene type were statistically significant, as has been reported previously [23, 24]. The caffeine-by-time interaction term also showed a small, but statistically significant (*p*-value = 0.007), positive association with ln(htTKV), whereas the main effect of caffeine in this model was not statistically significant (*p*-value = 0.205). In models with mGFR over time as the outcome, age, BMI and hypertension were the only statistically significant measures in our single factor analyses.

Next we fit multivariable models for ln(htTKV) and mGFR adjusted for age, sex, race, BMI, smoking, hypertension, gene type and time (Model 1). As with the single factor associations over time, age and hypertension were found to be statistically significant in Model 1 for both ln(htTKV) and mGFR (Additional file 2: Table S3). Gene type was also statistically significant for Model 1 for both outcomes (Additional file 2: Table S3). BMI was not statistically significant in Model 1.

Finally, we added caffeine consumption (any vs. none) to the multivariable models (Model 2) for both outcomes. These results are presented in Table 2. For both outcomes, the variables that were significant in Model 1 remained significant after the inclusion of caffeine consumption. The effect of caffeine on ln(htTKV) varied over time as indicated by tests of interactions between caffeine and time (*p*-value = 0.007) but not for mGFR (p-value = 0.811). The expected difference in ln(htTKV) between caffeine consumers and non-caffeine consumers at baseline was − 0.146 (95% CI: -0.295, 0.003; p-value = 0.061). This corresponds to a 13.6% (95% CI: 0.3, 25.5%) lower baseline htTKV associated with caffeine consumption. The expected difference in the rate of change of ln(htTKV) over time between caffeine consumers and non-caffeine consumers was 0.006 (95% CI: 0.002, 0.011; *p* = 0.007). This corresponds to a 0.6% (95% CI: 0.2, 1.1%) greater rate of kidney growth each year associated with caffeine, so the annual rate of kidney growth for caffeine consumers was 5.3% compared to 4.6% for non-caffeine consumers. The difference in baseline mGFR associated with caffeine consumption was 1.40 mL/min/1.73m^2^ (95% CI: -5.91, 8.71; *p* = 0.713) and the difference in the rate of change of mGFR was 0.069 mL/min/1.73m^2^ (95% CI: -0.495, 0.631; *p* = 0.811).

**Table 2:** Results from multivariable Model 2 (adjusted for caffeine) for ln(htTKV) and for mGFR

Fixed Effects	Ln(htTKV)			mGFR		
	Estimate	95% CI	*P*-value	Estimate	95% CI	*P*-value
Age	0.017	0.010, 0.025	< 0.001	−1.362	− 1.700, − 1.024	< 0.001
Sex (Male)	0.084	− 0.040, 0.208	0.195	−0.990	−6.757, 4.780	0.741
Race (White)	0.083	−0.101, 0.268	0.386	−0.811	−9.529, 7.880	0.858
BMI	0.006	−0.006, 0.018	0.345	−0.454	−1.001, 0.094	0.112
Smoke (Yes)	0.072	−0.091, 0.236	0.393	3.474	−4.111, 11.053	0.378
Hypertension (Yes)	0.395	0.263, 0.527	< 0.001	−11.946	−18.060, −5.821	< 0.001
Gene type			< 0.001*			0.018*
PKD1 + truncation	Reference	–		Reference	–	
PKD1 + no truncation	− 0.082	− 0.227, 0.064		1.475	− 5.263, 8.208	
PKD2 + NMD	−0.504	− 0.664, − 0.344		10.840	3.427, 18.237	
Caffeine (Any)	−0.146	−0.295, 0.003	0.061	1.395	−5.908, 8.714	0.713
Caffeine:Time (Any)	0.006	0.002, 0.011	0.007	0.069	−0.495, 0.631	0.811
Time	0.045	0.041, 0.049	< 0.001	−2.696	−3.187, −2.200	< 0.001
Random Effect		Variance	Standard Deviation		Variance	Standard Deviation
Patient		0.224	0.474		425.0	20.62
Residual		0.016	0.127		372.8	19.31

*P-value based on F-test with 3 groups. NMD refers to no mutation detected in the genetic analysis

### Caffeine intake and the risk of ESRD or death

**Fig. 1:**
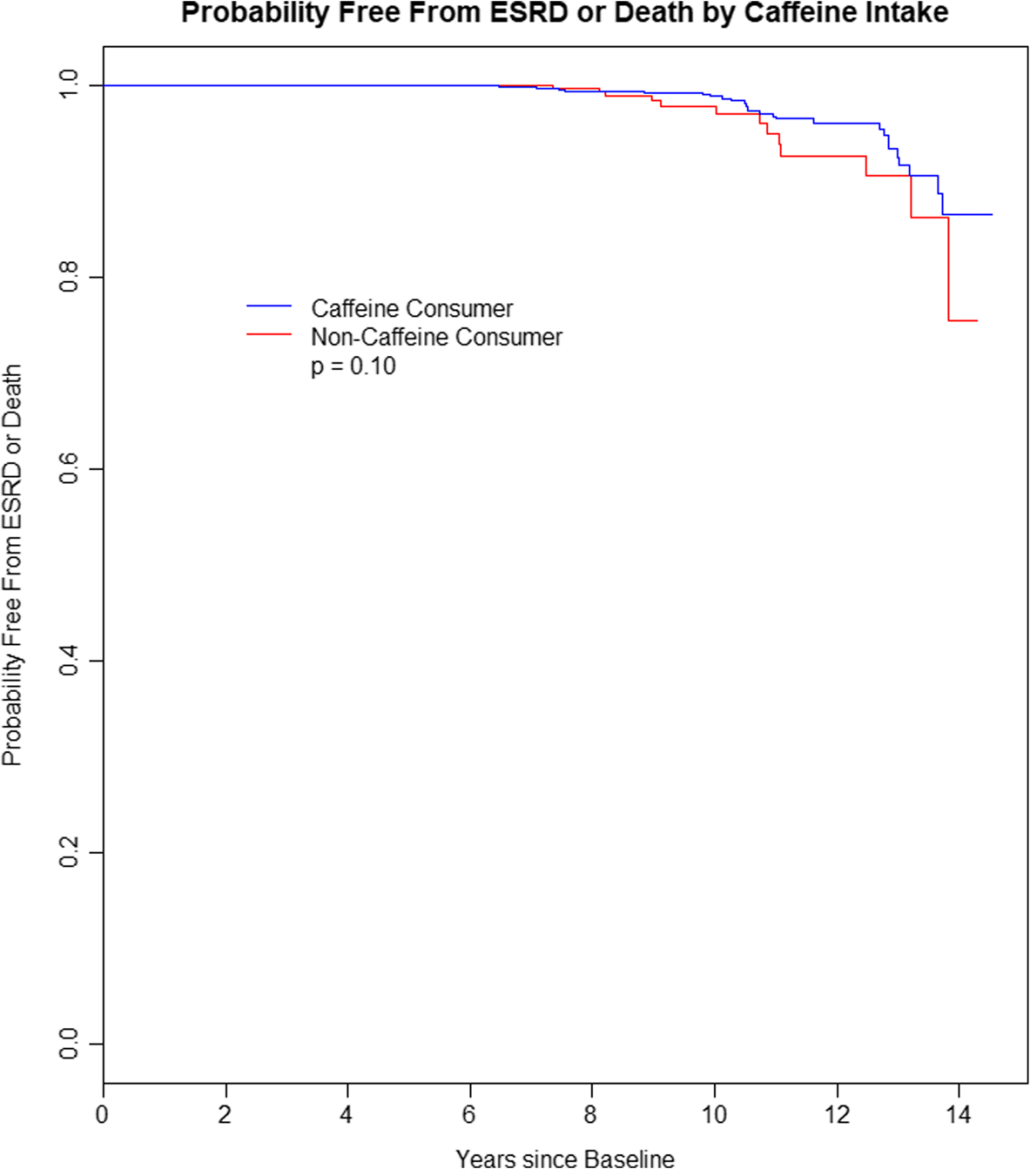
Kaplan Meier plot of probability free from ESRD or death according to caffeine intake

Among caffeine consumers, 15.4% reached ESRD and 1.1% died during the study, while in non-caffeine consumers, 25.5% reached ESRD and 1.2% died. The Kaplan-Meier curves for survival free of ESRD or death (Fig. 1) were not statistically significantly different (log rank test *p* = 0.10). In a multivariable proportional hazards model adjusted for the effects of age, sex, race, BMI, smoking, hypertension and gene type on time until ESRD or death (Additional file 2: Table S11), we found that age, smoking, hypertension and genetic status were statistically significant. When caffeine consumption (any vs. none) was added to this model, age, smoking, hypertension and gene type remained statistically significant (Table 3). There were no substantive changes in hazard for any of these variables. However, caffeine consumption was not found to be a statistically significant risk factor for the time to ESRD or death (Hazard Ratio (HR) = 0.556; 95% CI: 0.279, 1.110; *p* = 0.096).

**Table 3:** Cox Regression model with caffeine (any vs. none)

Risk Factor	Estimate	*P*-value	Hazard Ratio (HR) (95% CI for HR)
Age	0.079	< 0.001	1.083 (1.034, 1.133)
Sex (Male)	0.175	0.583	1.191 (0.638, 2.224)
Race (White)	− 1.030	0.069	0.357 (0.117, 1.085)
BMI	0.047	0.131	1.048 (0.986, 1.114)
Smoke (Yes)	1.042	0.017	2.836 (1.204, 6.683)
Hypertension (Yes)	1.528	0.003	4.611 (1.711, 12.426)
Gene type^a^			
PKD1 + no truncation	0.375	0.273	1.455 (0.745, 2.841)
PKD2 + NMD	−1.636	0.010	0.195 (0.057, 0.672)
Caffeine (Any)	−0.587	0.096	0.556 (0.279, 1.110)

^a^Reference group: PKD1 + truncation. NMD refers to no mutation detected in the genetic analysis

### Sensitivity analyses

We performed sensitivity analyses using different measures of amount of caffeine consumed examined as continuous and multicategory variables. We also generated these results excluding patients who were missing baseline data on both sources of caffeine (cups of coffee/tea and glasses of other caffeinated beverages). Both unadjusted and adjusted models were estimated. See Additional file 2: Tables S1-S10. When modeling ln(htTKV) as the outcome, caffeine:time interactions were statistically significant in most models but with estimates near zero. When modeling mGFR as the outcome, caffeine was not statistically significant. Therefore, the sensitivity analyses were not qualitatively different than our primary results and showed that caffeine does not have a strong and consistent effect on disease progression.

## Discussion

**Fig. 2:**
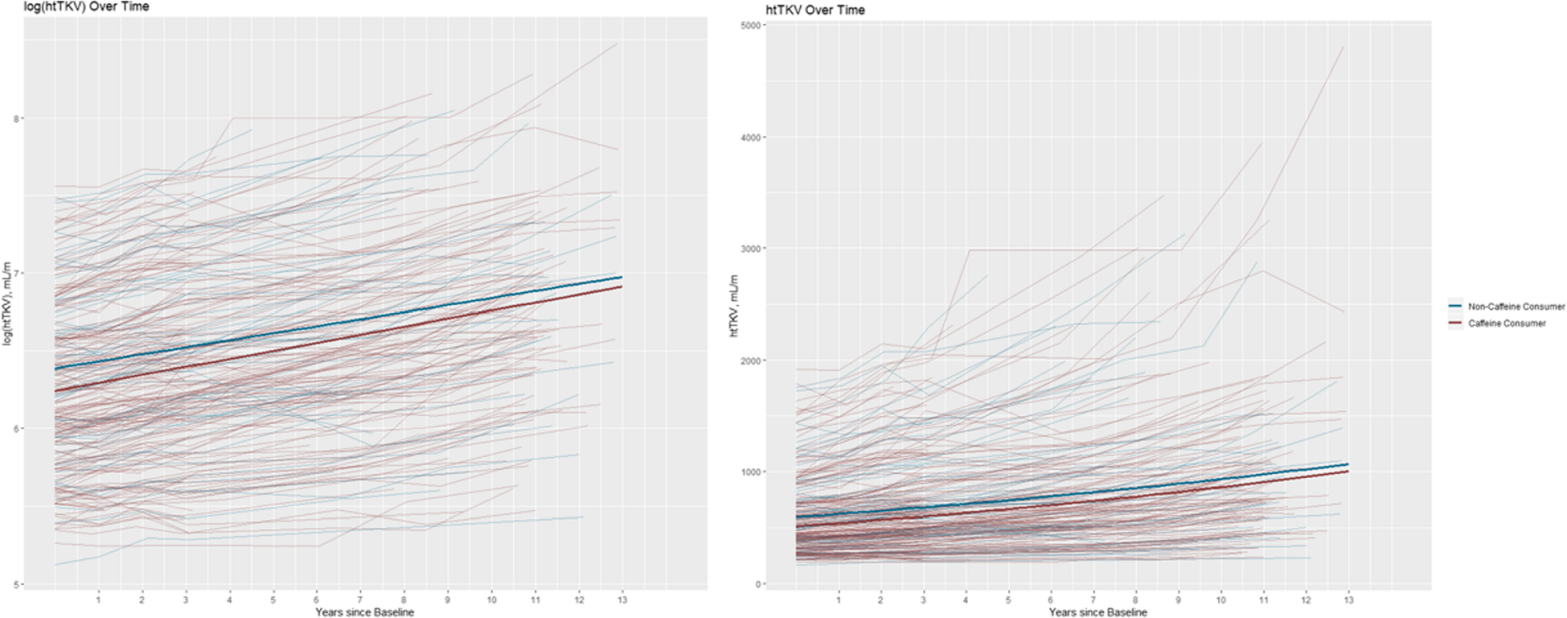
Spaghetti plot of ln(htTKV) (on left) and htTKV (on right) over time for each individual patient (randomly jittered to preclude presentation of any individual’s actual data). The final adjusted model is overlayed (Model 2), showing the differences in slope and intercept for patients who reported consuming caffeine and patients who did not report consuming caffeine at baseline

**Fig. 3:**
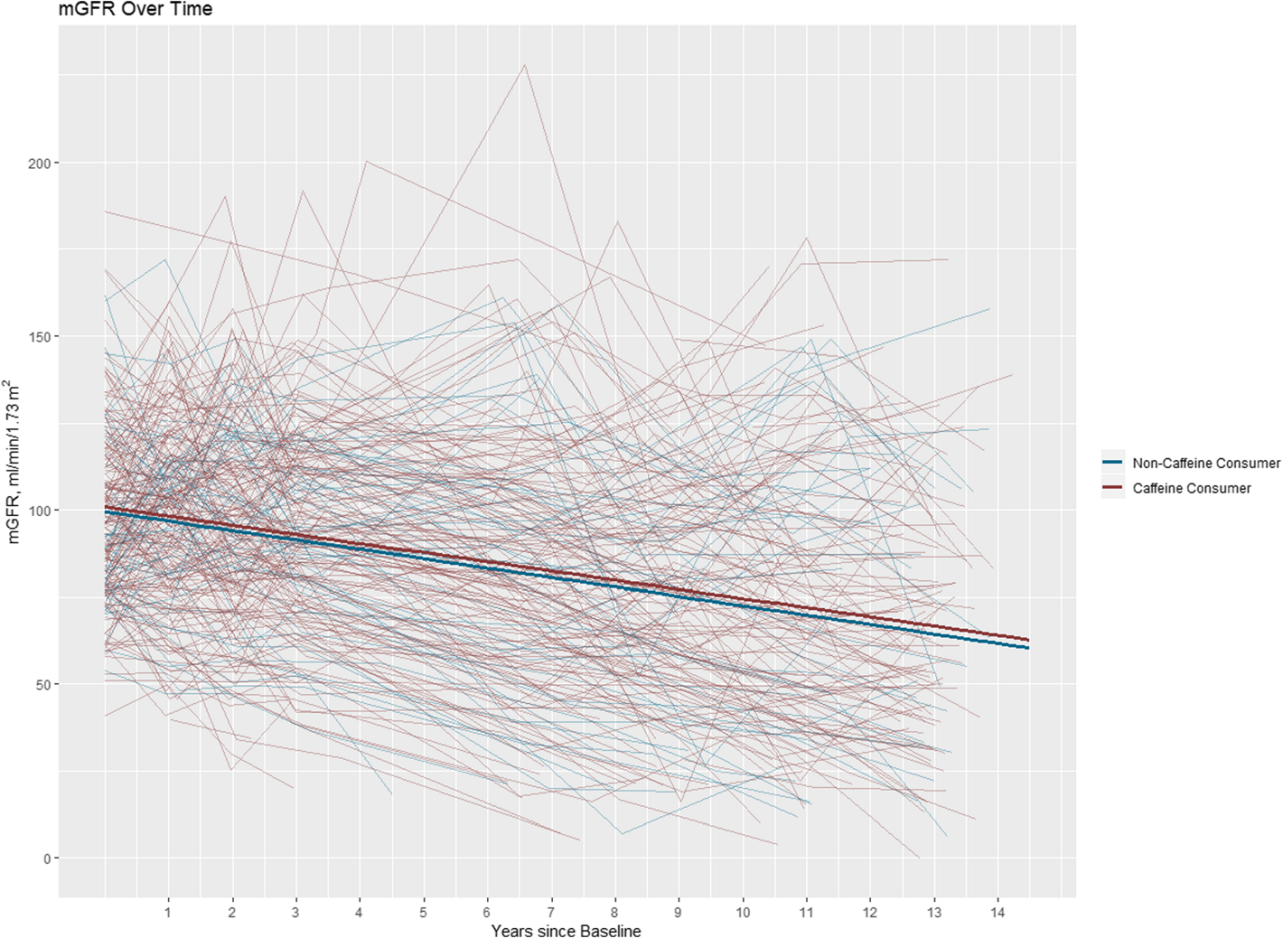
Spaghetti plot of mGFR over time for each individual patient (randomly jittered to preclude presentation of any individual’s actual data). The final adjusted model is overlayed (Model 2), showing the differences in slope and intercept for patients who reported consuming caffeine and patients who did not report consuming caffeine at baseline

In this analysis using data from the CRISP study, we found a statistically significant association between the interaction of time with caffeine (any vs. none) and ln(htTKV). This interaction term was positive, indicating that the rate of ln(htTKV) growth is higher with caffeine intake. However, the effect size of the interaction was quantitatively very small: 0.6% per year difference in the htTKV among caffeine consumers. Compared to the 4.6% average annual rate of increase in kidney size in non-caffeine consumers, the increased rate of 5.3% due to caffeine was small and unlikely to be clinically important. While the rate of kidney growth in caffeine consumers was higher, the expected htTKV at baseline and throughout the follow-up period showed a trend to be lower than for patients who did not consume caffeine (Fig. 2). Similar to the expected htTKV being lower throughout the follow-up period, the expected mGFR at baseline and throughout the follow-up period was higher for patients who consumed caffeine, although these results were not statistically significant (Fig. 3). The lack of association between caffeine and mGFR has been reported previously, including a meta-analysis that examined coffee consumption and chronic kidney disease in nearly 15,000 individuals [25]. These relationships were generally consistent throughout our sensitivity analyses. Taken together, these findings indicate that caffeine consumption is unlikely to have a clinically significant effect on ADPKD progression.

The reason why caffeine intake did not accelerate disease progression is unclear. One possible reason might be that the amount of caffeine that reaches the kidney instead of being metabolized in the liver may simply be too small [26]. Only 3% of caffeine is excreted intact [27]. Theoretically it is possible for caffeine to affect renal epithelial cAMP, but only in the patients with very high caffeine intake and high excretion rates of unmetabolized caffeine. In our analysis, patients in the highest category of caffeine intake did not have increased rates of kidney growth or GFR decline. In the majority of caffeine consumers, tissue exposure to caffeine may be too low to significantly increase cAMP in collecting duct cells and thus increase cyst and kidney volume. Additionally, this study examined caffeine intake at baseline only. Since patients with ADPKD are advised to limit caffeine intake, the patients in this study may have consumed less caffeine on average than what was reported at baseline. This could further decrease the amount of caffeine reaching the kidneys intact.

Another possible reason is because of the presumed natriuretic effects of caffeine [28]. Caffeine has been reported to be a potential acute inhibitor of sodium and hence water reabsorption in the proximal convoluted tubule [29]. If this were to be compensated by increased water intake, serum sodium concentration and osmolarity would tend to decrease thus suppressing vasopressin secretion. Vasopressin is believed to accelerate ADPKD progression by acting on V2 vasopressin receptors in cyst epithelial cells to increase cAMP. Tolvaptan, the only approved therapy for ADPKD, is a V2 receptor antagonist and has been shown to slow the increase in kidney volume [30]. As such, caffeine might have a beneficial effect on cellular cAMP levels through suppressing vasopressin levels that might counteract some or all of its effects on phosphodiesterases. However, no studies have shown the effects of caffeine intake on serum sodium concentration, urine osmolarity, urine volume and body fluid parameters in patients with chronic kidney disease. It is also important to note that studies completed on healthy adults have shown no effect of caffeine on these values [31, 32].

Finally, the effect of caffeine may have been masked by the presence of hypertension. Hypertension was a significant risk factor for disease progression in all of our models, and it is well known that acute consumption of caffeine increases blood pressure [33]. The relationship between chronic consumption of caffeine and hypertension is less clear. In some studies, chronic consumption of caffeine did not increase the frequency or severity of hypertension while in others, including one specifically studying ADPKD rats, it was associated with worsening of hypertension [9, 21, 34, 35]. Additionally, it is believed that caffeine exerts a variable response on hypertension due to genetic differences [36]. Thus it is possible that caffeine may exacerbate the deleterious effects of hypertension on ADPKD progression.

There are several limitations of this study. We assessed caffeine intake at baseline. Although caffeine intake is thought to be habitual and is not expected to change over time, some patients in our sample reported varying caffeine intake over time. This could be for many reasons. In 2015, KDIGO Guidelines included a formal recommendation for patients with ADPKD to avoid caffeine intake [8]. While this formal recommendation most likely did not impact this study because this data was collected before 2015, these guidelines reflect the general tendency to recommend that patients with ADPKD restrict caffeine intake. While these factors may have contributed to the results of this study, by analyzing only the baseline caffeine values, we emulated the clinical situation in which information might be limited to a single snapshot of the patient’s environmental and lifestyle exposures.

Another limitation is that CRISP was an observational prospective cohort study and the lifestyle data was self-reported. Additionally, caffeine content from food and the variability of caffeine content in beverages was not recorded in the CRISP questionnaire. As our primary analysis examined caffeine consumption as any vs. none, it may be limited by not including caffeine sources beyond coffee, tea and soft drinks. Finally, because the patients in CRISP were enrolled when they had relatively preserved GFR, the average rate of GFR decline is slow and few patients have reached ESRD so far, thus limiting the power to detect the effect of caffeine consumption on these outcomes.

Our study has several important strengths. CRISP is the largest and longest cohort study of ADPKD with a follow-up time of 14 years. This gave us the opportunity to explore the longitudinal relationship between caffeine and ADPKD progression, as measured by both htTKV and mGFR, over an extended timeframe. Another strength of this study was the availability of information on the intake of caffeinated beverages other than coffee and tea. This is important because beverages such as sodas constitute a significant source of dietary caffeine, particularly in the U.S. population. Finally, all the patients in this study were genotyped for PKD1 and PKD2 mutations, allowing us to adjust for the confounding effect of the PKD genes and allelic effects on both outcomes.

The most important strength of this study is that it used data from the CRISP cohort. The only other longitudinal study of caffeine and ADPKD progression was the study completed by Girardat-Rotar et al. in the Swiss ADPKD cohort [11]. While this study agreed with the conclusions of the Swiss ADPKD study, there are several key distinctions. First, this study had a sample size of 239 compared to the Swiss ADPKD sample size of 151. Second, the Swiss ADPKD study had a median follow-up time of 4.4 years while this study had a median follow-up time of 12.5 years. Third, the Swiss ADPKD study examined only coffee consumption as a caffeine source while this study included caffeinated beverages beyond coffee. Lastly, the Swiss study did not adjust for genotype while this study was able to utilize genetic information.

## Conclusions

In summary, we did not find compelling evidence that caffeine had a clinically significant detrimental effect on disease progression in patients with ADPKD. These conclusions are consistent with recent publications including the Girardat-Rotar et al. study [11] and a meta-analysis [25] – all concluding that caffeine was not associated with chronic kidney disease. Current recommendations to avoid caffeine exposure in ADPKD are not supported by this and other clinical evidence.

## Additional files

Additional file 1: Survey questions asked of patients concerning their environmental exposures, specifically regarding caffeine consumption. (PDF 231 kb)

Additional file 2: Description of all models and results from sensitivity analyses. (DOCX 102 kb)

## Abbreviations

ADPKD: Autosomal dominant polycystic kidney disease
cAMP: 3′:5′-cyclic adenosine monophosphate
CFTR: Cystic fibrosis transmembrane conductance regulator
CRISP: Consortium for Radiologic Imaging Studies of Polycystic Kidney Disease
ESRD: End stage renal disease
htTKV: Height adjusted total kidney volume
mGFR: Iothalamate clearance
NMD: No mutation detected
