# Supplementary material for: Relationship between caffeine intake and autosomal dominant polycystic kidney disease progression: a retrospective analysis using the CRISP cohort
Source: BMC Nephrol. 2018 Dec 27;19:378. doi: 10.1186/s12882-018-1182-0 (PMC6307167; doi:10.1186/s12882-018-1182-0)
Supplement: Supplementary file 1 — Survey questions asked of patients concerning their environmental exposures, specifically regarding caffeine consumption. (PDF 231 kb) [file 12882_2018_1182_MOESM1_ESM.pdf]

CRISP III

Attention - DO NOT enter patient data on this form if the header does not contain *preprinted* CRISP ID number, clinical center ID, and visit number.

Participant ID:_____________ *pkdid* **Clinical Center**: __________ *pccn*
visit:

Missing Data Codes:	A-Participant Refused	B-Reading Not Possible	C-Institutional Error

Biannual Clinic Visit/Meds and Events

This form is to be completed at Visits 10 and 12.

1. **Date of visit** *dvdate* [ ][ ] / [ ][ ] / [ ][ ][ ][ ]

2. **Since last contact, has the participant had any illnesses?** *ilyn* 0 ☐ No **(*Go to #3)*** 1 ☐ Yes

If yes, **please specify briefly:** *ill*____

____

____

2a. **Have you been newly diagnosed with hypertension since last contact?** *hypert* 0 ☐ No 1 ☐ Yes

If yes, **Date of diagnosis:** __ __/__ __ /__ __ __ __ *hypyr*
hypmt *hypda* Month Day Year

How were you diagnosed with hypertension? *hyphdia* 1 ☐ Home BP monitor 2 ☐ Doctor visit
3 ☐ Hospital stay 4 ☐ Other Specify: __________________ *hypspc*

2b. **Since last contact, have you been told by a doctor or other health professional that you have diabetes or high blood sugar (If female, other than during pregnancy)?** *diablowsugar*

0 ☐ No 1 ☐ Yes 2 ☐ Don't Know 9998 ☐ Missing

If no or don't know, and male participant, go to 3.
If no or don't know, and female participant, go to 2c.

If yes, date: Month [ ][ ] / Day [ ][ ] / Year [ ][ ][ ][ ] *diablowsugarmt* *diablowsugarda* *diablowsugaryr*

If male participant, go to 2d.

2c. **Since last contact, have you been told by a doctor or other health professional that you have/had diabetes during pregnancy?** *diabpregn*

0 ☐ No 1 ☐ Yes 2 ☐ Don't Know 9998 ☐ Missing

If 2c=no or don't know, go to 3.

If yes, date: Month [ ][ ] / Day [ ][ ] / Year [ ][ ][ ][ ] *diabpregnmt* *diabpregnda* *diabpregnyr*

2d. **Since last contact, have you taken medications for diabetes?** *diabmed*

0 ☐ No 1 ☐ Yes 2 ☐ Don't Know 777 ☐ Not Applicable 9998 ☐ Missing

Attention - DO NOT enter patient data on this form if the header does not contain *preprinted* CRISP ID number, clinical center ID, and visit number.

Participant ID:_____________ *pkdid* **Clinical Center**: __________ *pccn*
visit:

Missing Data Codes:	A-Participant Refused	B-Reading Not Possible	C-Institutional Error

Biannual Clinic Visit/Meds and Events

If yes, medications:
_________________________ *diabmed1*
_________________________ *diabmed2*
_________________________ *diabmed3*
_________________________ *diabmed4*
_________________________ *diabmed5*

**2e.**	**Since last contact, have you taken diabetic pills (also called hypoglycemic agents) to lower your blood sugar?** *diabpills*	0 ☐ No	1 ☐ Yes	2 ☐ Don't Know	777 ☐ Not Applicable	9998 ☐ Missing
**2f.**	**Since last contact, have you taken insulin?** *insulin*	0 ☐ No	1 ☐ Yes	2 ☐ Don't Know	777 ☐ Not Applicable	9998 ☐ Missing

3. **Since the last contact, has the participant visited their primary care physician?** *pvyn* 0 ☐ No **(*Go to #4*)** 1 ☐ Yes

If yes, complete Section 3

3a. Date of physician visit: __ __/__ __ /__ __ __ __*pvyr*
pvmt *pvda* Month Day Year

3b. Were there multiple visits to this physician? *Mvci* 0 ☐ No 1 ☐ Yes

3c. Name and address of physician treating participant:

Name: ____

Address: ____

City, State, Zip: ____

3d. Specify reason for visit: *pvreason* ____

____

____

____

Attention - DO NOT enter patient data on this form if the header does not contain *preprinted* CRISP ID number, clinical center ID, and visit number.

Participant ID:______________ *pkdid* **Clinical Center**: ___________ *pccn*
visit:

Missing Data Codes: A-Participant Refused B-Reading Not Possible C-Institutional Error

Biannual Clinic Visit/Meds and Events

4. Since last contact, has the participant visited any physician other than the primary care physician listed in question 3? *pvotphyId* 0 ☐ No ***(Go to #5)*** 1 ☐ Yes

If yes, complete Section #4

Physician #1

a. Date of additional physician visit: __ __/__ __ /__ __ __ __ *pv2yr*
pv2mt *pv2da* Month Day Year

b. Were there multiple visits to this physician? *m2vc* 0 ☐ No 1 ☐ Yes

c. Name and address of physician treating participant:

Name: ____

Address: ____

City, State, Zip:____

d. Specify reason for visit: *pv2reason*____

____

____

Physician #2

a. Date of additional physician visit: __ __/__ __ /__ __ __ __ *pv2yr*
pv2mt *pv2da* Month Day Year

b. Were there multiple visits to this physician? *m2vc* 0 ☐ No 1 ☐ Yes

c. Name and address of physician treating participant:

Name: ____

Address: ____

City, State, Zip:____

d. Specify reason for visit: *pv2reason*____

____

____

Missing Data Codes:	A-Participant Refused	B-Reading Not Possible	C-Institutional Error

Biannual Clinic Visit/Meds and Events

Physician #3

a. Date of additional physician visit: __ __/__ __/__ __ __ __ *pv2yr*
pv2mt *pv2da* Month Day Year

b. Were there multiple visits to this physician? *m2vc* 0 ☐ No 1 ☐ Yes

c. Name and address of physician treating participant:

Name: ____

Address: ____

City, State, Zip:____

d. Specify reason for visit: *pv2reason*____

____

____

Physician #4

a. Date of additional physician visit: __ __/__ __/__ __ __ __ *pv2yr*
pv2mt *pv2da* Month Day Year

b. Were there multiple visits to this physician? *m2vc* 0 ☐ No 1 ☐ Yes

c. Name and address of physician treating participant:

Name: ____

Address: ____

City, State, Zip:____

d. Specify reason for visit: *pv2reason*____

____

____

Attention - DO NOT enter patient data on this form if the header does not contain *preprinted* CRISP ID number, clinical center ID, and visit number.

Participant ID:_____________ *pkdid* **Clinical Center**: __________ *pccn*
visit:

Missing Data Codes:	A-Participant Refused	B-Reading Not Possible	C-Institutional Error

Biannual Clinic Visit/Meds and Events

Physician #5

a. Date of additional physician visit: __ __/__ __/__ __ __ __ *pv2yr*
pv2mt *pv2da* Month Day Year

b. Were there multiple visits to this physician? *m2vc* 0 ☐ No 1 ☐ Yes

c. Name and address of physician treating participant:

Name: ______________________________

Address: ______________________________

City, State, Zip:______________________________

d. Specify reason for visit: *pv2reason*______________________________

____

____

Physician #6

a. Date of additional physician visit: __ __/__ __/__ __ __ __ *pv2yr*
pv2mt *pv2da* Month Day Year

b. Were there multiple visits to this physician? *m2vc* 0 ☐ No 1 ☐ Yes

c. Name and address of physician treating participant:

Name: ______________________________

Address: ______________________________

City, State, Zip:______________________________

d. Specify reason for visit: *pv2reason*______________________________

____

____

Missing Data Codes:	A-Participant Refused	B-Reading Not Possible	C-Institutional Error

Biannual Clinic Visit/Meds and Events

Physician #7

a. Date of additional physician visit: __ __/__ __ /__ __ __ __ *pv2yr*
pv2mt *pv2da* Month Day Year

b. Were there multiple visits to this physician? *m2vc* 0 ☐ No 1 ☐ Yes

c. Name and address of physician treating participant:

Name: ______________________________

Address: ______________________________

City, State, Zip: ______________________________

d. Specify reason for visit: *pv2reason* ______________________________

____

____

Physician #8

a. Date of additional physician visit: __ __/__ __ /__ __ __ __ *pv2yr*
pv2mt *pv2da* Month Day Year

b. Were there multiple visits to this physician? *m2vc* 0 ☐ No 1 ☐ Yes

c. Name and address of physician treating participant:

Name: ______________________________

Address: ______________________________

City, State, Zip: ______________________________

d. Specify reason for visit: *pv2reason* ______________________________

____

____

Biannual Clinic Visit/Meds and Events

Physician #9

a. Date of additional physician visit: __ __/__ __/__ __ __ __ *pv2yr*
pv2mt *pv2da* Month Day Year

b. Were there multiple visits to this physician? *m2vc* 0 ☐ No 1 ☐ Yes

c. Name and address of physician treating participant:

Name: ____________________

Address: ____________________

City, State, Zip:____________________

d. Specify reason for visit: *pv2reason*____________________

____

____

Physician #10

a. Date of additional physician visit: __ __/__ __/__ __ __ __ *pv2yr*
pv2mt *pv2da* Month Day Year

b. Were there multiple visits to this physician? *m2vc0* 0 ☐ No 1 ☐ Yes

c. Name and address of physician treating participant:

Name: ____________________

Address: ____________________

City, State, Zip:____________________

d. Specify reason for visit: *pv2reason*____________________

____

____

Please continue on the next page

Missing Data Codes: A-Participant Refused B-Reading Not Possible C-Institutional Error

Biannual Clinic Visit/Meds and Events

5. Since last contact, has the participant been hospitalized? *hospId* 0 ☐ No **(*Go to #6)*** 1 ☐ Yes

If yes, complete Section #5

Hospitalization #1

a. Was this hospitalization unscheduled? *husch* 0 ☐ No 1 ☐ Yes **(See Note)**

.

Note: If unscheduled, please report the event to the local IRB and send a copy to the DCIAC

b. Date admitted to hospital: __ __/__ __/__ __ __ __ *hayr*
hamt *hada* Month Day Year

c. Date discharged from hospital: __ __/__ __/__ __ __ __ *hdyr*
hdmt *hdda* Month Day Year

d. Length of stay (in days) : ________*lenst*

e. Name and address of hospital:

Name:____

Address:____

City, State, Zip:____

f. Name and address of physician treating participant:

Name:____

Address:____

City, State, Zip:____

g. What was the discharge diagnosis?______________________________*hdiag*

h. Was there any renal surgery performed? *rsurgpyn* 0 ☐ No 1 ☐ Yes
If no, go to Hospitalization #2 or Section 6 if no more hospitalizations

If yes, was the intent cyst reduction? *ceducyn* 0 ☐ No 1 ☐ Yes

i. For any renal surgery provide a date and short description:
Date of intervention: __ __/__ __/__ __ __ __ *rsiyr*
rsimt *rsida* Month Day Year

Description:____*rsidesc*

Missing Data Codes: A-Participant Refused B-Reading Not Possible C-Institutional Error

Biannual Clinic Visit/Meds and Events

Hospitalization #2

a. Was this hospitalization unscheduled? *husch* 0 ☐ No 1 ☐ Yes **(See Note)**

.

Note: If unscheduled, please report the event to the local IRB and send a copy to the DCIAC

b. Date admitted to hospital: __ __/__ __/__ __ __ __ *hayr*
hamt *hada* Month Day Year

c. Date discharged from hospital: __ __/__ __/__ __ __ __ *hdyr*
hdmt *hdda* Month Day Year

d. Length of stay (in days) : ________*lenst*

e. Name and address of hospital:

Name:____

Address:____

City, State, Zip:____

f. Name and address of physician treating participant:

Name:____

Address:____

City, State, Zip:____

g. What was the discharge diagnosis?________________________________*hdiag*

h. Was there any renal surgery performed? *resurgpyn* 0 ☐ No 1 ☐ Yes
If no, go to Hospitalization #3 or Section 6 if no more hospitalizations

If yes, was the intent cyst reduction? *ceducyn* 0 ☐ No 1 ☐ Yes

i. For any renal surgery provide a date and short description:

Date of intervention: __ __/__ __/__ __ __ __ *rsiyr*
rsimt *rsida* Month Day Year

Description:____*rsidesc*

Attention - DO NOT enter patient data on this form if the header does not contain *preprinted* CRISP ID number, clinical center ID, and visit number.

Participant ID:_____________ *pkdid* **Clinical Center**: __________ *pccn*
visit:

Missing Data Codes:	A-Participant Refused	B-Reading Not Possible	C-Institutional Error

Biannual Clinic Visit/Meds and Events

Hospitalization #3

a. Was this hospitalization unscheduled? *husch* 0 ☐ No 1 ☐ Yes **(See Note)**

.

Note: If unscheduled, please report the event to the local IRB and send a copy to the DCIAC

b. Date admitted to hospital: __ __/__ __/__ __ __ __ *hayr*
hamt *hada* Month Day Year

c. Date discharged from hospital: __ __/__ __/__ __ __ __ *hdyr*
hdmt *hdda* Month Day Year

d. Length of stay (in days) : ________*lenst*

e. Name and address of hospital:

Name:____

Address:____

City, State, Zip:____

f. Name and address of physician treating participant:

Name:____

Address:____

City, State, Zip:____

g. What was the discharge diagnosis?____*hdiag*

h. Was there any renal surgery performed? *rsurgpyn* 0 ☐ No 1 ☐ Yes
If no, go to Hospitalization #4 or Section 6 if no more hospitalizations

If yes, was the intent cyst reduction? *ceducyn* 0 ☐ No 1 ☐ Yes

i. For any renal surgery provide a date and short description:

Date of intervention: __ __/__ __/__ __ __ __ *rsiyr*
rsimt *rsida* Month Day Year

Description:____*rsidesc*

Attention - DO NOT enter patient data on this form if the header does not contain *preprinted* CRISP ID number, clinical center ID, and visit number.

Participant ID:_____________ *pkdid* **Clinical Center**: __________ *pccn*
visit:

Missing Data Codes: A-Participant Refused B-Reading Not Possible C-Institutional Error

Biannual Clinic Visit/Meds and Events

Hospitalization #4

a. Was this hospitalization unscheduled? *husch* 0 ☐ No 1 ☐ Yes **(See Note)**

.

Note: If unscheduled, please report the event to the local IRB and send a copy to the DCIAC

b. Date admitted to hospital: __ __/__ __/__ __ __ __ *hayr*
hamt *hada* Month Day Year

c. Date discharged from hospital: __ __/__ __/__ __ __ __ *hdyr*
hdmt *hdda* Month Day Year

d. Length of stay (in days) : ________*lenst*

e. Name and address of hospital:

Name:____

Address:____

City, State, Zip:____

f. Name and address of physician treating participant:

Name:____

Address:____

City, State, Zip:____

g. What was the discharge diagnosis?____*hdiag*

h. Was there any renal surgery performed? *rsurgpyn* 0 ☐ No ***(Go to #6)*** 1 ☐ Yes

If yes, was the intent cyst reduction? *ceducyn* 0 ☐ No 1 ☐ Yes

i. For any renal surgery provide a date and short description:

Date of intervention: __ __/__ __/__ __ __ __ *rsiyr*
rsimt *rsida* Month Day Year

Description:____*rsidesc*

Attention - DO NOT enter patient data on this form if the header does not contain *preprinted* CRISP ID number, clinical center ID, and visit number.

Participant ID:______________ *pkdid* **Clinical Center**: ___________ *pccn*
visit:

Missing Data Codes: A-Participant Refused B-Reading Not Possible C-Institutional Error

Biannual Clinic Visit/Meds and Events

6. Smoking and Tobacco:

6a. Has the participant ever smoked cigarettes? *csyn* 0 ☐ No **(*Go to# 6e)*** 1 ☐ Yes

6b. *If yes,* *csevsm*
- 1 ☐ Current ***(Go to #6d)***
- 2 ☐ Former, quit since last visit ***(Go to #6c)***
- 3 ☐ Former, quit prior to last visit ***(Go to #6c)***

6c. If former smoker, quit date: __ __/__ __ __ __ ***(Go to #6e)***
qsm Month Year *qsy*

6d. If current smoker, how many packs per year does the participant smoke? *ppy*
__ __ __ __

6e. Has the participant used any other types of tobacco since last contact? *otytob* 0 ☐ No **(*Go to #7a)*** 1 ☐ Yes

6f. *If yes*, **which types?**

6g. Cigars 0 ☐ No 1 ☐ Yes *cigar*

6h. *If yes*, **how many cigars since the last contact? __ __** *cignm*

6i. Pipe 0 ☐ No 1 ☐ Yes *pipeyn*

6j. Chewing Tobacco/Snuff 0 ☐ No 1 ☐ Yes *chewyn*

7. Caffeinated Beverages:

7a. Does the participant drink caffeinated coffee or tea? *cucaff* 0 ☐ No **(Go to #7b)** 1 ☐ Yes

If yes, check time interval and enter the average number of caffeinated 8 ounce cups per Interval: *cupcaf*

- 0 ☐ Per day
- 1 ☐ Per week
- 2 ☐ Per month

Number of 8 ounce cups per interval__ __ *ccafunit*

Attention - DO NOT enter patient data on this form if the header does not contain *preprinted* CRISP ID number, clinical center ID, and visit number.

Participant ID:_____________ *pkdid* **Clinical Center**: __________ *pccn*
visit:

Missing Data Codes: A-Participant Refused B-Reading Not Possible C-Institutional Error

Biannual Clinic Visit/Meds and Events

7b. Does the participant drink other caffeinated beverages? *cafotbv* 0 ☐ No **(*Go to #7c*)** 1 ☐ Yes

If yes, check time interval and enter the average number of caffeinated 12 ounce portions per interval: *glassc*

0 ☐ Per day
1 ☐ Per week **Number of 12 ounce portions per interval** __ __ *scafunit*
2 ☐ Per month

7c. Does the participant drink alcohol? *alcdr* 0 ☐ No **(*Go to #8*)** 1 ☐ Yes

If yes, check time interval and enter the average number of alcoholic drinks per interval: *nad*

(1 drink=any of the following: 12 ounces of beer, 4 ounces of wine, 1.5 ounces liquor)
0 ☐ Per day
1 ☐ Per week **Number of drinks per interval** __ __ *alconit*
2 ☐ Per month

8. Analgesic Use History: *Record the average number per month over the last year. 0=Participant doesn't use*

8a. Acetaminophen tablets: __ __ __ *acett* (Avg. number per month)
8b. Aspirin Tablets: __ __ __ *asprt* (Avg. number per month)

8c. Combination analgesics: __ __ __ *combot* (Avg. number per month)
8d. NSAIDs: __ __ __ *nsaidt* (Avg. number per month)

8e. Medical use of marijuana: __ __ __ *dum* (Avg. Number per month)
8f. Cox2 Inhibitors __ __ __ *cox2* (Avg. number per month)

9. Has the participant used recreational drugs in the last year? *illdrg* 0 ☐ No 1 ☐ Yes

If yes, check all that apply
☐ Heroin *duh*
☐ Marijuana *duma*
☐ Methamphetamine *dumeth*
☐ Cocaine *duc*
☐ Other *duo*

If other, specify:______________________________*othr*

Attention - DO NOT enter patient data on this form if the header does not contain *preprinted* CRISP ID number, clinical center ID, and visit number.

Participant ID:______________ *pkdid* **Clinical Center**: ___________ *pccn*
visit:

Missing Data Codes:	A-Participant Refused	B-Reading Not Possible	C-Institutional Error

Biannual Clinic Visit/Meds and Events

If this is Visit 10 and the participant is in Crisp II, or if this is Visit 12, Go to # 11.

If this is Visit 10 and the participant is in Crisp I only,

10. List all current prescription medications, over the counter medications and all natural products/protein supplements,

and then ***STOP***

Prescribed Medications *presId*

pres

Over the Counter Medications *octId*

oct

All Natural Products/ Protein Supplements *nppId*

npp

Attention - DO NOT enter patient data on this form if the header does not contain *preprinted* CRISP ID number, clinical center ID, and visit number.

Participant ID:______________ *pkdid* **Clinical Center**: ___________ *pccn*
visit:

Missing Data Codes: A-Participant Refused B-Reading Not Possible C-Institutional Error

Biannual Clinic Visit/Meds and Events

11. Prescribed medications changes:

11a. Since last contact, have prescribed drugs been added? *pmald*

If yes, then please record:

Prescribed Medications **added**	Date (month/year)
pma	*dpmamt* __ __/__ __ __ __ *dpmayr*

CRISP III

Attention - DO NOT enter patient data on this form if the header does not contain *preprinted* CRISP ID number, clinical center ID, and visit number.

Participant ID:______________ *pkdid* **Clinical Center**: ___________ *pccn*
visit:

Missing Data Codes: A-Participant Refused B-Reading Not Possible C-Institutional Error

Biannual Clinic Visit/Meds and Events

11b. Since last contact, have prescribed drugs been stopped/discontinued?
pmdId
If yes, then please record:

Prescribed Medications **discontinued**	Date (month/year)
pmd	*dpmdmt* __ __/__ __ __ __ *dpmdyr*

Biannual Clinic Visit/Meds and Events

12. Over-the-counter medications changes:

12a. Since last contact, have OTC drugs been added? *omald*

If yes, then please record:

OTC Medications **added**	Date (month/year)
oma	*domamt* __ __/__ __ __ __ *domayr*

12b. Since last contact, have OTC drugs been stopped/discontinued? *omdld*

If yes, then please record:

OTC Medications **discontinued**	Date (month/year)
omd	*domdmt* __ __/__ __ __ __ *domdyr*

Attention - DO NOT enter patient data on this form if the header does not contain *preprinted* CRISP ID number, clinical center ID, and visit number.

Participant ID:______________ *pkdid* **Clinical Center**: ___________ *pccn*
visit:

Missing Data Codes:	A-Participant Refused	B-Reading Not Possible	C-Institutional Error

Biannual Clinic Visit/Meds and Events

13. Natural Product Use Changes:

13a. Since last contact, have Natural Products/Protein Supplements been added? *nmald*

If yes, then please record:

Natural Products/Protein Supplements **added**	Date (month/year)
nma	*dnmamt* __ __/__ __ __ __ *dnmayr*

13b. Since last contact, have Natural Products/Protein Supplements been stopped/discontinued?
nmdld
If yes, then please record:

Natural Products/Protein Supplements **discontinued**	Date (month/year)
nmd	*dnmdmt* __ __/__ __ __ __ *dnmdyr*

Please review all contact information on the Identification Form including phone number and email address.

CRISP Member completing this form_______________________
cdidnum

Date Form Completed __ __/__ __/__ __ __ __
cddate

Data Entry Status: Please check to indicate that the above information has been entered ☐

Primary Entered by: _______________________________ Date: __ __/__ __/__ __ __ __ *dedate*
deidnum

Secondary Entered by: ____________________________ Date __ __/__ __/__ __ __ __
