# Supplementary material for: Relationship between caffeine intake and autosomal dominant polycystic kidney disease progression: a retrospective analysis using the CRISP cohort
Source: BMC Nephrol. 2018 Dec 27;19:378. doi: 10.1186/s12882-018-1182-0 (PMC6307167; doi:10.1186/s12882-018-1182-0)
Supplement: Supplementary file 2 — Description of all models and results from sensitivity analyses. (DOCX 102 kb) [file 12882_2018_1182_MOESM2_ESM.docx]

**Additional file 1: Table S1. Description of models created during this analysis.**

| Classification | Outcome | Fixed Effects | Random Effect | R Code |
| --- | --- | --- | --- | --- |
| Single Factor Associations Over Time Without Adjustment | Ln(htTKV) and mGFR | Age + Time | Subject | lmer(outcome ~ age + time + (1\|pkdid), data = finalData1) |
|  | Ln(htTKV) and mGFR | Sex + Time | Subject | lmer(outcome ~ sex + time + (1\|pkdid), data = finalData1) |
|  | Ln(htTKV) and mGFR | Race + Time | Subject | lmer(outcome ~ race + time + (1\|pkdid), data = finalData1) |
|  | Ln(htTKV) and mGFR | BMI + Time | Subject | lmer(outcome ~ bmi + time + (1\|pkdid), data = finalData1) |
|  | Ln(htTKV) and mGFR | Smoking + Time | Subject | lmer(outcome ~ smoke + time + (1\|pkdid), data = finalData1) |
|  | Ln(htTKV) and mGFR | Hypertension + Time | Subject | lmer(outcome ~ hyt + time + (1\|pkdid), data = finalData1) |
|  | Ln(htTKV) and mGFR | Gene type + Time | Subject | lmer(outcome ~ genetype + time + (1\|pkdid), data = finalData1) |
|  | Ln(htTKV) and mGFR | Caffeine + Time + Caffeine:Time | Subject | lmer(outcome ~ caffeine*time + (1\|pkdid), data = finalData1) |
| Model 1 (Multivariable Adjusted) | Ln(htTKV) and mGFR | Age + Sex + Race + BMI + Smoking + Hypertension + Gene type + Time | Subject | lmer(outcome ~ age + sex + race + bmi + smoke + hyt + genetype + time + (1\|pkdid), data = finalData1) |
| Model 2 (Multivariable Adjusted with Caffeine) | Ln(htTKV) and mGFR | Age + Sex + Race + BMI + Smoking + Hypertension + Gene type + Time + Caffeine + Caffeine:Time | Subject | lmer(outcome ~ age + sex + race + bmi + smoke + hyt + genetype + time*caffeine + (1\|pkdid), data = finalData1) |
| Survival Analysis (Baseline) | Time to ESRD/Death | Age + Sex + Race + BMI + Smoking + Hypertension + Gene type | NA | coxph(Surv(time = test1$time, event = test1$event, type = "right")~ age + sex + race + bmi + smoke + hyt + genetype, data = test1, model = TRUE) |
| Survival Analysis (with caffeine) | Time to ESRD/Death | Age + Sex + Race + BMI + Smoking + Hypertension + Gene type + Caffeine | NA | coxph(Surv(time = test1$time, event = test1$event, type = "right")~ age + sex + race + bmi + smoke + hyt + genetype + caffeine, data = test1, model = TRUE) |

**Additional file 1: Table S2. Results from single factor associations over time without adjustment.**

| Variable | | | ln(htTKV) | | | mGFR | | |
| --- | --- | --- | --- | --- | --- | --- | --- | --- |
|  |  |  | Estimate | 95% CI | P-value | Estimate | 95% CI | P-value |
| Age | | | 0.020 | 0.012, 0.028 | < 0.001 | -1.507 | -1.843, -1.171 | < 0.001 |
|  | Time | | 0.050 | 0.048, 0.0522 | < 0.001 | -2.643 | -2.881, -2.405 | < 0.001 |
| Sex (Male) | | | 0.081 | -0.071, 0.233 | 0.296 | -1.274 | -8.233, 5.685 | 0.720 |
|  | Time | | 0.050 | 0.048, 0.052 | < 0.001 | -2.644 | -2.882, -2.406 | < 0.001 |
| Race (White) | | | 0.285 | 0.069, 0.501 | 0.0104 | -5.623 | -15.728, 4.489 | 0.277 |
|  | Time | | 0.050 | 0.048, 0.052 | < 0.001 | -2.642 | -2.880, -2.404 | < 0.001 |
| BMI | | | 0.018 | 0.004, 0.032 | 0.011 | -1.145 | -1.777, -0.514 | < 0.001 |
|  | | Time | 0.050 | 0.048, 0.052 | < 0.001 | -2.644 | -2.882, -2.406 | < 0.001 |
| Smoking (Yes) | | | -0.036 | -0.236, 0.163 | 0.721 | 6.493 | -2.618, 15.604 | 0.164 |
|  | | Time | 0.050 | 0.048, 0.052 | < 0.001 | -2.64 | -2.879, -2.403 | < 0.001 |
| Hypertension (Yes) | | | 0.537 | 0.401, 0.674 | < 0.001 | -21.113 | -27.588, -14.643 | < 0.001 |
|  | Time | | 0.050 | 0.048, 0.052 | < 0.001 | -2.647 | -2.884, -2.409 | < 0.001 |
| Gene type | | |  |  | < 0.001* |  |  | 0.290* |
|  | PKD1 + truncation | | Reference | --- |  | Reference | --- |  |
|  | PKD1 + no truncation | | -0.051 | -0.222, 0.120 |  | -0.920 | -9.138, 7.298 |  |
|  | PKD2 + NMD | | -0.469 | -0.649, -0.289 |  | 6.279 | -2.364, 14.923 |  |
|  | Time | | 0.050 | 0.048, 0.052 | < 0.001 | -2.644 | -2.882, -2.405 | < 0.001 |
| Caffeine (Any) | | | -0.118 | -0.300, 0.064 | 0.205 | 1.333 | -7.352, 10.017 | 0.764 |
|  | Time | | 0.045 | 0.041, 0.049 | < 0.001 | -2.697 | -3.192, -2.202 | < 0.001 |
|  | Caffeine:Time** | | 0.007 | 0.002, 0.011 | 0.007 | 0.070 | -0.495, 0.634 | 0.809 |
| Caffeine (mg) | | | 0.006 | -0.066, 0.078 | 0.870 | 0.979 | -2.462, 4.418 | 0.578 |
|  | Time | | 0.050 | 0.048, 0.052 | < 0.001 | -2.645 | -2.883, -2.406 | < 0.001 |
|  | Caffeine:Time | | -0.002 | -0.004, -0.0004 | 0.019 | -0.024 | -0.281, 0.233 | 0.855 |
| Caffeine (Multicategory) | | |  |  | 0.610*** |  |  | 0.923 |
|  | No Caffeine | | Reference | --- |  | Reference | --- |  |
|  | 1^st^ Quartile | | -0.178 | -0.408, 0.051 |  | -1.037 | -11.983, 9.908 |  |
|  | 2^nd^ Quartile | | -0.139 | -0.372, 0.094 |  | 4.102 | -7.034, 15.239 |  |
|  | 3^rd^ Quartile | | -0.083 | -0.319, 0.152 |  | 0.856 | -10.398, 12.110 |  |
|  | 4^th^ Quartile | | -0.066 | -0.300, 0.168 |  | 1.625 | -9.581, 12.833 |  |
|  | Caffeine:time | |  |  | < 0.001*** |  |  | 0.550 |
|  | No Caffeine:Time | | Reference | --- |  | Reference | --- |  |
|  | 1^st^ Quartile:Time | | 0.014 | 0.008, 0.020 |  | 0.453 | -0.266, 1.171 |  |
|  | 2^nd^ Quartile:Time | | 0.015 | 0.009, 0.022 |  | -0.075 | -0.817, 0.669 |  |
|  | 3^rd^ Quartile:Time | | -0.003 | -0.009, 0.003 |  | 0.015 | -0.734, 0.765 |  |
|  | 4^th^ Quartile:Time | | 0.001 | -0.005, 0.007 |  | -0.146 | -0.874, 0.580 |  |
|  | Time | | 0.045 | 0.041, 0.049 | < 0.001 | -2.698 | -3.191, -2.203 | < 0.001 |

*P-value based on F-test with 3 groups. **Caffeine:Time represents the caffeine by time interaction.
*** P-value based on F-test with 5 groups.

**Additional file 1: Table S3. Results from multivariable Model 1 (not adjusted for caffeine) for ln(htTKV) and for mGFR.**

| Fixed Effects | | Ln(htTKV) | | | mGFR | | |
| --- | --- | --- | --- | --- | --- | --- | --- |
|  |  | Estimate | 95% CI | P-value | Estimate | 95% CI | P-value |
| Age | | 0.017 | 0.010, 0.025 | < 0.001 | -1.361 | -1.699, -1.023 | < 0.001 |
| Sex (Male) | | 0.079 | -0.046, 0.204 | 0.224 | -0.929 | -6.693, 4.840 | 0.756 |
| Race (White) | | 0.066 | -0.118, 0.251 | 0.488 | -0.581 | -9.246, 8.063 | 0.897 |
| BMI | | 0.005 | -0.007, 0.017 | 0.407 | -0.443 | -0.989, 0.103 | 0.119 |
| Smoke (Yes) | | 0.056 | -0.106, 0.219 | 0.505 | 3.706 | -3.826, 11.232 | 0.343 |
| Hypertension (Yes) | | 0.401 | 0.269, 0.533 | < 0.001 | -12.045 | -18.152, -5.928 | < 0.001 |
| Gene type | |  |  | < 0.001* |  |  | 0.019* |
|  | PKD1 + truncation | Reference | --- |  | Reference | --- |  |
|  | PKD1 + no truncation | -0.084 | -0.231, 0.062 |  | 1.492 | -5.248, 8.228 |  |
|  | PKD2 + NMD | -0.502 | -0.662, -0.341 |  | 10.775 | 3.362, 18.173 |  |
| Time | | 0.050 | 0.048, 0.052 | < 0.001 | -2.643 | -2.879, -2.404 | < 0.001 |
|  | |  |  |  |  |  |  |
| Random Effect | |  | Variance | Standard Deviation |  | Variance | Standard Deviation |
| Patient | |  | 0.226 | 0.475 |  | 423.5 | 20.58 |
| Residual | |  | 0.016 | 0.127 |  | 372.6 | 19.30 |

*P-value based on F-test with 3 groups.

**Additional file 1: Table S4. Results from Model 2 for ln(htTKV) and Model 2 for mGFR, using caffeine as a continuous variable (mg).**

| Fixed Effects | | Ln(htTKV) | | | mGFR | | |
| --- | --- | --- | --- | --- | --- | --- | --- |
|  |  | Estimate | 95% CI | P-value | Estimate | 95% CI | P-value |
| Age | | 0.017 | 0.010, 0.025 | < 0.001 | -1.364 | -1.702, -1.027 | < 0.001 |
| Sex (Male) | | 0.080 | -0.047, 0.203 | 0.231 | -0.854 | -6.613, 4.908 | 0.775 |
| Race (White) | | 0.071 | -0.114, 0.256 | 0.459 | -0.819 | -9.494, 7.830 | 0.856 |
| BMI | | 0.005 | -0.007, 0.017 | 0.399 | -0.445 | -0.990, 0.101 | 0.118 |
| Smoke (Yes) | | 0.066 | -0.099, 0.232 | 0.439 | 3.142 | -4.501, 10.780 | 0.429 |
| Hypertension (Yes) | | 0.401 | 0.269, 0.533 | < 0.001 | -12.060 | -18.156, -5.951 | < 0.001 |
| Gene type | |  |  | < 0.001* |  |  | 0.018* |
|  | PKD1 + truncation | Reference | --- |  | Reference | --- |  |
|  | PKD1 + no truncation | -0.089 | -0.236, 0.057 |  | 1.761 | -5.002, 8.518 |  |
|  | PKD2 + NMD | -0.504 | -0.664, -0.344 |  | 10.920 | 3.510, 18.314 |  |
| Caffeine (mg) | | -0.012 | -0.071, 0.047 | 0.693 | 1.225 | -1.680, 4.136 | 0.417 |
| Caffeine:Time (mg) | | -0.002 | -0.004, 0.000 | 0.019 | -0.026 | -0.282, 0.230 | 0.845 |
| Time | | 0.050 | 0.048, 0.052 | < 0.001 | -2.643 | -2.880, -2.404 | < 0.001 |
|  | |  |  |  |  |  |  |
| Random Effect | |  | Variance | Standard Deviation |  | Variance | Standard Deviation |
| Patient | |  | 0.226 | 0.475 |  | 424.1 | 20.59 |
| Residual | |  | 0.016 | 0.127 |  | 372.8 | 19.31 |

*P-value based on F-test with 3 groups. Caffeine (mg) was centered.

**Additional file 1: Table S5. Results from Model 2 for ln(htTKV) and Model 2 for mGFR, using caffeine as a multicategory variable.**

| Fixed Effects | | Ln(htTKV) | | | mGFR | | |
| --- | --- | --- | --- | --- | --- | --- | --- |
|  |  | Estimate | 95% CI | P-value | Estimate | 95% CI | P-value |
| Age | | 0.019 | 0.011, 0.026 | < 0.001 | -1.418 | -1.759, -1.078 | < 0.001 |
| Sex (Male) | | 0.079 | -0.045, 0.202 | 0.224 | -0.629 | -6.355, 5.106 | 0.834 |
| Race (White) | | 0.098 | -0.086, 0.282 | 0.308 | -0.924 | -9.618, 7.725 | 0.839 |
| BMI | | 0.004 | -0.007, 0.016 | 0.469 | -0.403 | -0.949, 0.143 | 0.159 |
| Smoke (Yes) | | 0.057 | -0.108, 0.223 | 0.506 | 3.448 | -4.227, 11.113 | 0.391 |
| Hypertension (Yes) | | 0.411 | 0.279, 0.543 | < 0.001 | -12.694 | -18.795, -6.573 | < 0.001 |
| Gene type | |  |  | < 0.001* |  |  | 0.018* |
|  | PKD1 + truncation | Reference | --- |  | Reference | --- |  |
|  | PKD1 + no truncation | -0.097 | -0.242, 0.049 |  | 1.969 | -4.787, 8.716 |  |
|  | PKD2 + NMD | -0.500 | -0.659, -0.342 |  | 10.898 | 3.533, 18.239 |  |
| Caffeine (Multicategory) | |  |  | 0.232** |  |  | 0.338** |
|  | No Caffeine | Reference | --- |  | Reference | --- |  |
|  | 1^st^ Quartile | -0.161 | -0.346, 0.024 |  | -3.065 | -12.169, 6.061 |  |
|  | 2^nd^ Quartile | -0.088 | -0.278, 0.103 |  | 0.089 | -9.279, 9.468 |  |
|  | 3^rd^ Quartile | -0.221 | -0.412, -0.031 |  | 7.235 | -2.141, 16.638 |  |
|  | 4^th^ Quartile | -0.102 | -0.294, 0.090 |  | 1.490 | -7.947, 10.956 |  |
| Caffeine:Time | |  |  | < 0.001** |  |  | 0.568** |
|  | No Caffeine:Time | Reference | --- |  | Reference | --- |  |
|  | 1^st^ Quartile:Time | 0.014 | 0.008, 0.020 |  | 0.445 | -0.272, 1.161 |  |
|  | 2^nd^ Quartile:Time | 0.016 | 0.009, 0.022 |  | -0.066 | -0.806, 0.676 |  |
|  | 3^rd^ Quartile:Time | -0.003 | -0.009, 0.003 |  | 0.003 | -0.745, 0.751 |  |
|  | 4^th^ Quartile:Time | 0.001 | -0.005, 0.007 |  | -0.140 | -0.867, 0.582 |  |
| Time | | 0.045 | 0.041, 0.049 | < 0.001 | -2.696 | -3.187, -2.200 | < 0.001 |
|  | |  |  |  |  |  |  |
| Random Effect | |  | Variance | Standard Deviation |  | Variance | Standard Deviation |
| Patient | |  | 0.223 | 0.473 |  | 422.9 | 20.56 |
| Residual | |  | 0.015 | 0.124 |  | 372.9 | 19.31 |

*P-value based on F-test with 3 groups. **P-value based on F-test with 5 groups. Details on how each quartile of caffeine intake was formed can be found in the Methods.

**Additional file 1: Table S6. Results from single factor associations over time without adjustment and excluding subjects with missing caffeine values at baseline (n = 223).**

| Variable | | | ln(htTKV) | | | mGFR | | |
| --- | --- | --- | --- | --- | --- | --- | --- | --- |
|  |  |  | Estimate | 95% CI | P-value | Estimate | 95% CI | P-value |
| Age | | | 0.019 | 0.011, 0.028 | < 0.001 | -1.502 | -1.858, -1.146 | < 0.001 |
|  | Time | | 0.051 | 0.049, 0.053 | < 0.001 | -2.656 | -2.901, -2.409 | < 0.001 |
| Sex (Male) | | | 0.080 | -0.077, 0.237 | 0.320 | -0.806 | -7.944, 6.329 | 0.825 |
|  | Time | | 0.051 | 0.049, 0.053 | < 0.001 | -2.655 | -2.902, -2.408 | < 0.001 |
| Race (White) | | | 0.308 | 0.077, 0.538 | 0.010 | -7.437 | -18.131, 3.265 | 0.174 |
|  | Time | | 0.051 | 0.049, 0.053 | < 0.001 | -2.653 | -2.900, -2.406 | < 0.001 |
| BMI | | | 0.017 | 0.002, 0.031 | 0.023 | -1.074 | -1.726, -0.423 | 0.001 |
|  | | Time | 0.051 | 0.049, 0.053 | < 0.001 | -2.655 | -2.901, -2.408 | < 0.001 |
| Smoke (Yes) | | | -0.058 | -0.262, 0.146 | 0.579 | 7.860 | -1.334, 17.055 | 0.095 |
|  | | Time | 0.051 | 0.049, 0.053 | < 0.001 | -2.652 | -2.898, -2.405 | < 0.001 |
| Hypertension (Yes) | | | 0.540 | 0.400, 0.682 | < 0.001 | -21.878 | -28.473, -15.288 | < 0.001 |
|  | Time | | 0.051 | 0.049, 0.053 | < 0.001 | -2.661 | -2.907, -2.414 | < 0.001 |
| Gene type | | |  |  | < 0.001* |  |  | 0.217* |
|  | PKD1 + truncation | | Reference | --- |  | Reference | --- |  |
|  | PKD1 + no truncation | | -0.064 | -0.238, 0.110 |  | 0.003 | -8.312, 8.319 |  |
|  | PKD2 + NMD | | -0.503 | -0.691, -0.315 |  | 7.686 | -1.319, 16.693 |  |
|  | Time | | 0.051 | 0.049, 0.053 | < 0.001 | -2.655 | -2.901, -2.407 | < 0.001 |
| Caffeine (Any) | | | -0.114 | -0.327, 0.100 | 0.298 | 0.038 | -10.028, 10.101 | 0.994 |
|  | Time | | 0.049 | 0.044, 0.054 | < 0.001 | -2.792 | -3.393, -2.189 | < 0.001 |
|  | Caffeine:Time** | | 0.003 | -0.003, 0.008 | 0.330 | 0.164 | -0.496, 0.824 | 0.626 |
| Caffeine (mg) | | | 0.013 | -0.062, 0.087 | 0.737 | 0.800 | -2.740, 4.338 | 0.658 |
|  | Time | | 0.051 | 0.049, 0.053 | < 0.001 | -2.655 | -2.902, -2.408 | < 0.001 |
|  | Caffeine:Time | | -0.004 | -0.006, -0.001 | < 0.001 | -0.015 | -0.282, 0.252 | 0.913 |
| Caffeine (Multicategory) | | |  |  |  |  |  |  |
|  | No Caffeine | | Reference | --- | 0.705* | Reference | --- | 0.935* |
|  | 1^st^ Quartile | | -0.174 | -0.429, 0.081 |  | -2.333 | -14.378, 9.711 |  |
|  | 2^nd^ Quartile | | -0.135 | -0.393, 0.123 |  | 2.807 | -9.410, 15.024 |  |
|  | 3^rd^ Quartile | | -0.079 | -0.339, 0182 |  | -0.440 | -12.762, 11.883 |  |
|  | 4^th^ Quartile | | -0.061 | -0.320, 0.198 |  | 0.330 | -11.949, 12.611 |  |
|  | No Caffeine:Time | | Reference | --- | < 0.001* | Reference | --- | 0.516* |
|  | 1^st^ Quartile:Time | | 0.010 | 0.003, 0.017 |  | 0.547 | -0.247, 1.341 |  |
|  | 2^nd^ Quartile:Time | | 0.012 | 0.005, 0.018 |  | 0.020 | -0.796, 0.837 |  |
|  | 3^rd^ Quartile:Time | | -0.007 | -0.014, -0.001 |  | 0.110 | -0.713, 0.933 |  |
|  | 4^th^ Quartile:Time | | -0.003 | -0.009, 0.004 |  | -0.051 | -0.855, 0.749 |  |
|  | Time | | 0.049 | 0.044, 0.054 | < 0.001 | -2.792 | -3.392, -2.190 | < 0.001 |

*P-value based on F-test with 3 groups.

**Additional file 1: Table S7. Results from Model 1 for ln(htTKV) and Model 1 for mGFR, excluding subjects with missing caffeine values at baseline (n = 223).**

| Fixed Effects | | Ln(htTKV) | | | mGFR | | |
| --- | --- | --- | --- | --- | --- | --- | --- |
|  |  | Estimate | 95% CI | P-value | Estimate | 95% CI | P-value |
| Age | | 0.016 | 0.008, 0.024 | < 0.001 | -1.341 | -1.697, -0.984 | < 0.001 |
| Sex (Male) | | 0.085 | -0.046, 0.217 | 0.211 | -1.356 | -7.332, 4.624 | 0.662 |
| Race (White) | | 0.068 | -0.131, 0.267 | 0.508 | -0.913 | -10.153, 8.299 | 0.849 |
| BMI | | 0.004 | -0.009, 0.016 | 0.585 | -0.391 | -0.953, 0.172 | 0.182 |
| Smoke (Yes) | | 0.046 | -0.121, 0.213 | 0.597 | 4.868 | -2.766, 12.497 | 0.220 |
| Hypertension (Yes) | | 0.404 | 0.264, 0.544 | < 0.001 | -12.500 | -18.861, -6.127 | < 0.001 |
| Gene type | |  |  | < 0.001* |  |  | 0.031* |
|  | PKD1 + truncation | Reference | --- |  | Reference | --- |  |
|  | PKD1 + no truncation | -0.092 | -0.242, 0.057 |  | 1.923 | -4.899, 8.739 |  |
|  | PKD2 + NMD | -0.509 | -0.679, -0.339 |  | 10.628 | 2.890, 18.350 |  |
| Time | | 0.051 | 0.049, 0.053 | < 0.001 | -2.656 | -2.900, -2.408 | < 0.001 |
|  | |  |  |  |  |  |  |
| Random Effect | |  | Variance | Standard Deviation |  | Variance | Standard Deviation |
| Patient | |  | 0.231 | 0.480 |  | 420.1 | 20.50 |
| Residual | |  | 0.016 | 0.125 |  | 369.4 | 19.22 |

*P-value based on F-test with 3 groups.

**Additional file 1: Table S8. Results from Model 2 for ln(htTKV) and Model 2 for mGFR, excluding subjects with missing caffeine values at baseline (n = 223).**

| Fixed Effects | | Ln(htTKV) | | | mGFR | | |
| --- | --- | --- | --- | --- | --- | --- | --- |
|  |  | Estimate | 95% CI | P-value | Estimate | 95% CI | P-value |
| Age | | 0.016 | 0.008, 0.024 | < 0.001 | -1.342 | -1.699, -0.985 | < 0.001 |
| Sex (Male) | | 0.085 | -0.046, 0.216 | 0.212 | -1.356 | -7.331, 4.625 | 0.663 |
| Race (White) | | 0.081 | -0.119, 0.280 | 0.437 | -0.834 | -10.121, 8.420 | 0.863 |
| BMI | | 0.004 | -0.009, 0.016 | 0.550 | -0.390 | -0.952, 0.174 | 0.185 |
| Smoke (Yes) | | 0.057 | -0.111, 0.114 | 0.517 | 4.911 | -2.764, 12.579 | 0.220 |
| Hypertension (Yes) | | 0.402 | 0.263, 0.542 | < 0.001 | -12.505 | -18.867, -6.130 | < 0.001 |
| Gene type | |  |  | < 0.001* |  |  | 0.031* |
|  | PKD1 + truncation | Reference | --- |  | Reference | --- |  |
|  | PKD1 + no truncation | -0.095 | -0.245, 0.054 |  | 1.936 | -4.892, 8.758 |  |
|  | PKD2 + NMD | -0.509 | -0.679, -0.340 |  | 10.651 | 2.911, 18.374 |  |
| Caffeine (Any) | | -0.119 | -0.293, 0.055 | 0.190 | -1.202 | -9.603, 7.215 | 0.783 |
| Caffeine:Time (Any) | | 0.003 | -0.003, 0.008 | 0.352 | 0.178 | -0.481, 0.835 | 0.597 |
| Time | | 0.049 | 0.044, 0.054 | < 0.001 | -2.804 | -3.402, -2.201 | < 0.001 |
|  | |  |  |  |  |  |  |
| Random Effect | |  | Variance | Standard Deviation |  | Variance | Standard Deviation |
| Patient | |  | 0.230 | 0.480 |  | 422.5 | 20.56 |
| Residual | |  | 0.016 | 0.125 |  | 369.6 | 19.23 |

*P-value based on F-test with 3 groups.

**Additional file 1: Table S9. Results from Model 2 for ln(htTKV) and Model 2 for mGFR, using caffeine as a continuous variable (mg), excluding subjects with missing caffeine values at baseline (n = 223).**

| Fixed Effects | | Ln(htTKV) | | | mGFR | | |
| --- | --- | --- | --- | --- | --- | --- | --- |
|  |  | Estimate | 95% CI | P-value | Estimate | 95% CI | P-value |
| Age | | 0.016 | 0.008, 0.024 | < 0.001 | -1.341 | -1.697, -0.985 | < 0.001 |
| Sex (Male) | | 0.084 | -0.047, 0.215 | 0.220 | -1.262 | -7.242, 4.723 | 0.685 |
| Race (White) | | 0.072 | -0.127, 0.271 | 0.489 | -1.065 | -10.317, 8.155 | 0.825 |
| BMI | | 0.004 | -0.009, 0.016 | 0.583 | -0.389 | -0.951, 0.174 | 0.185 |
| Smoke (Yes) | | 0.052 | -0.117, 0.222 | 0.553 | 4.499 | -3.243, 12.236 | 0.265 |
| Hypertension (Yes) | | 0.405 | 0.265, 0.544 | < 0.001 | -12.556 | -18.915, -6.185 | < 0.001 |
| Gene type | |  |  | < 0.001* |  |  | 0.030* |
|  | PKD1 + truncation | Reference | --- |  | Reference | --- |  |
|  | PKD1 + no truncation | -0.097 | -0.248, 0.054 |  | 2.160 | -4.711, 9.024 |  |
|  | PKD2 + NMD | -0.509 | -0.679, -0.340 |  | 10.717 | 2.977, 18.438 |  |
| Caffeine (mg) | | -0.002 | -0.065, 0.060 | 0.946 | 0.844 | -2.153, 3.845 | 0.588 |
| Caffeine:Time (mg) | | -0.004 | -0.006, -0.002 | < 0.001 | -0.015 | -0.282, 0.249 | 0.911 |
| Time | | 0.051 | 0.049, 0.053 | < 0.001 | -2.656 | -2.901, -2.408 | < 0.001 |
|  | |  |  |  |  |  |  |
| Random Effect | |  | Variance | Standard Deviation |  | Variance | Standard Deviation |
| Patient | |  | 0.231 | 0.481 |  | 421.7 | 20.53 |
| Residual | |  | 0.015 | 0.124 |  | 369.7 | 19.23 |

*P-value based on F-test with 3 groups. Caffeine (mg) was centered.

**Additional file 1: Table S10. Results from Model 2 for ln(htTKV) and Model 2 for mGFR, using caffeine represented as a multicategory variable, excluding subjects with missing caffeine values at baseline (n = 223).**

| Fixed Effects | | Ln(htTKV) | | | mGFR | | |
| --- | --- | --- | --- | --- | --- | --- | --- |
|  |  | Estimate | 95% CI | P-value | Estimate | 95% CI | P-value |
| Age | | 0.017 | 0.009, 0.025 | < 0.001 | -1.403 | -1.762, -1.044 | < 0.001 |
| Sex (Male) | | 0.080 | -0.050, 0.210 | 0.240 | -1.014 | -6.939, 4.923 | 0.745 |
| Race (White) | | 0.097 | -0.101, 0.296 | 0.350 | -0.986 | -10.243, 8.217 | 0.838 |
| BMI | | 0.002 | -0.001, 0.015 | 0.715 | -0.332 | -0.892, 0.231 | 0.261 |
| Smoke (Yes) | | 0.040 | -0.129, 0.210 | 0.651 | 4.958 | -2.804, 12.710 | 0.224 |
| Hypertension (Yes) | | 0.420 | 0.281, 0.560 | < 0.001 | -13.370 | -19.721, -6.998 | < 0.001 |
| Gene type | |  |  | < 0.001* |  |  | 0.032* |
|  | PKD1 + truncation | Reference | --- |  | Reference | --- |  |
|  | PKD1 + no truncation | -0.111 | -0.261, 0.039 |  | 2.472 | -4.371, 9.305 |  |
|  | PKD2 + NMD | -0.503 | -0.671, -0.336 |  | 10.639 | 2.953, 18.298 |  |
| Caffeine (Multicategory) | |  |  | 0.431** |  |  | 0.341** |
|  | No Caffeine | Reference | --- |  | Reference | --- |  |
|  | 1^st^ Quartile | -0.140 | -0.346, 0.067 |  | -5.412 | -15.388, 4.587 |  |
|  | 2^nd^ Quartile | -0.061 | -0.272, 0.149 |  | -2.670 | -12.857, 7.528 |  |
|  | 3^rd^ Quartile | -0.195 | -0.406, 0.017 |  | 4.815 | -5.423, 15.082 |  |
|  | 4^th^ Quartile | -0.074 | -0.287, 0.138 |  | -1.263 | -11.545, 9.049 |  |
| Caffeine:Time | |  |  | < 0.001** |  |  | 0.526** |
|  | No Caffeine:Time | Reference | --- |  | Reference | --- |  |
|  | 1^st^ Quartile:Time | 0.010 | 0.003, 0.016 |  | 0.556 | -0.237, 1.348 |  |
|  | 2^nd^ Quartile:Time | 0.012 | 0.005, 0.018 |  | 0.042 | -0.770, 0.858 |  |
|  | 3^rd^ Quartile:Time | -0.007 | -0.014, -0.001 |  | 0.113 | -0.708, 0.933 |  |
|  | 4^th^ Quartile:Time | -0.003 | -0.010, 0.003 |  | -0.028 | -0.830, 0.769 |  |
| Time | | 0.049 | 0.044, 0.054 | < 0.001 | -2.805 | -3.403, -2.203 | < 0.001 |
|  | |  |  |  |  |  |  |
| Random Effect | |  | Variance | Standard Deviation |  | Variance | Standard Deviation |
| Patient | |  | 0.229 | 0.479 |  | 419.9 | 20.49 |
| Residual | |  | 0.015 | 0.122 |  | 369.7 | 19.23 |

*P-value based on F-test with 3 groups. **P-value based on F-test with 5 groups. Details on how each quartile of caffeine intake was formed can be found in the Methods.

**Additional file 1: Table S11. Cox Regression baseline model.**

| Risk Factor | | Estimate | P-value | Hazard Ratio (HR)  (95% CI for HR) |
| --- | --- | --- | --- | --- |
| Age | | 0.077 | < 0.001 | 1.080 (1.033, 1.130) |
| Sex (Male) | | 0.209 | 0.514 | 1.232 (0.657, 2.309) |
| Race (White) | | -1.177 | 0.038 | 0.308 (0.101, 0.937) |
| BMI | | 0.038 | 0.213 | 1.039 (0.978, 1.103) |
| Smoke (Yes) | | 1.045 | 0.017 | 2.845 (1.202, 6.733) |
| Hypertension (Yes) | | 1.650 | 0.001 | 5.209 (1.945, 13.951) |
| Gene type* | |  |  |  |
|  | PKD1+no truncation | 0.418 | 0.216 | 1.519 (0.784, 2.944) |
|  | PKD2 + NMD | -1.605 | 0.012 | 0.201 (0.058, 0.698) |

*Reference group: PKD1 + truncation.
